# Obsessive-Compulsive Aspects and Pathological Gambling in an Italian Sample

**DOI:** 10.1155/2014/167438

**Published:** 2014-06-25

**Authors:** Filippo Petruccelli, Pierluigi Diotaiuti, Valeria Verrastro, Irene Petruccelli, Maria Luisa Carenti, Domenico De Berardis, Felice Iasevoli, Alessandro Valchera, Michele Fornaro, Giovanni Martinotti, Massimo Di Giannantonio, Luigi Janiri

**Affiliations:** ^1^Department of Human, Social and Health Sciences, University of Cassino, 03043 Cassino, Italy; ^2^Faculty of Human and Social Sciences, Kore University of Enna, 94100 Enna, Italy; ^3^Department of Neuroscience and Imaging, Institute of Psychiatry, G. d'Annunzio University of Chieti-Pescara, 66100 Chieti, Italy; ^4^Department of Neuroscience, Reproductive Sciences and Odontostomatology, Federico II University of Naples, 80138 Naples, Italy; ^5^Hermanas Hospitalarias, Villa San Giuseppe Hospital, 63100 Ascoli Piceno, Italy; ^6^Department of Educational Sciences, University of Catania, 95124 Catania, Italy; ^7^Institute of Psychiatry and Psychology, Catholic University of Rome, 00198 Rome, Italy

## Abstract

*Introduction.* Gambling behaviour appears as repetitive and difficult to resist and seems to be aimed at neutralizing or reducing negative feelings such as anxiety and tension, confirming its similarities with the obsessive-compulsive spectrum. *Aims.* Estimating the prevalence of gambling behaviour in an Italian sample and assessing the effects of sociodemographic variables and the correlations between gambling behaviour and obsessive-compulsive features. *Methods.* A sample of 300 Italian subjects was evaluated based on gambling behaviours and obsessive-compulsive attitudes. The assessment was carried out in small centers in Italy, mainly in coffee and tobacco shops, where slot machines are located, using the *South Oaks Gambling Screen* (SOGS) and the MOCQ-R, a reduced form of *Maudsley Obsessional-Compulsive Questionnaire*. *Results.* A negative correlation between SOGS and MOPQ-R, with reference to the *control* and *cleaning* subscales, was evidenced in the majority of the examined subjects. Both evaluating instruments showed reliability and a good discriminative capacity. *Conclusions.* Our study evidenced that the sample of gamblers we analysed did not belong to the obsessive-compulsive disorders area, supporting the validity of the model proposed by DSM-5 for the classification of PG. These data confirm the importance of investing in treatments similar to those used for substance use disorders.

## 1. Introduction

Pathological gambling (PG) is defined as a maladaptive and recurrent pattern of gambling behaviours that persists despite substantial negative consequences for the individual, his/her work, and his/her family [1]. This dysfunctional behaviour is frequently associated with increased financial, legal, and psychological problems [2–4]. The prevalence of this social practice in Italy is growing, as it has been estimated that 54% of the Italian adult population (between 18 and 74 years old) gambles at least once a year. There are about 30 million gamblers divided into various categories of games, and the use of money for gambling, betting, and raffling has increased from 6000 to 17000 million € in four years [5, 6]. PG has also been reported in adolescents, posing important issues regarding prevention [7]. Currently, DSM-5 includes PG in the addictive disorders category: it is named as gambling disorder (GD), and it is the only new addiction included, representing the only one “without a substance” [8]. GD shares many similarities with substance use disorders (SUDs), for example, the progressive loss of control over the behaviour, the search for a euphoric state or “high,” craving, tolerance, and withdrawal symptoms [8, 9]. The biological bases of PG have also been described, representing one of the reasons of its inclusion in the addiction section of DSM-5 [10–12]. Furthermore, but not less important, PG shows many similarities to obsessive-compulsive disorder (OCD) [13, 14]. Indeed, the debate on how to consider PG, whether as an addictive disorder or as a disorder belonging to the obsessive-compulsive spectrum, remained open until the publication of the new manual [15]. The concept of obsessive-compulsive related disorders was proposed by different researchers in the early 1990s and refers to a class of disorders that share features with OCD [16]. OCD is the prototype of compulsive disorders. Obsessions are defined as recurrent and persistent thoughts, perceived by the subject as intrusive. Compulsive behaviours are defined as repetitive, rigid, and stereotyped goal-directed action; individuals refer to being driven to perform them in order to prevent or reduce perceived negative consequences [17]. The preoccupation with gambling, described in the DSM-5, recalls the obsessive thoughts typically observed in patients who suffer from OCD [18]; moreover, the gambling behaviour appears as repetitive and difficult to resist, and seems aimed at neutralizing or reducing negative moods such as anxiety and tension, showing again similarities with OCD [16]. It has been hypothesized that compulsivity in addiction derives from a dysregulation of specific neurochemical elements, involved in reward and stress systems in brain. An allostatic process between a loss in reward function and the recruitment of brain stress system provides a powerful base for the development of negative states that contribute to compulsive behaviours (negative reward) [19, 20]. Another hypothesis proposes the involvement of the dimension of anhedonia in compulsive behaviour. The impairment of hedonic capacity, possibly resulting in an underlying neuropsychological dysfunction, may be decisive in determining the engagement in frequent and repeated episodes of gambling, which represent a compensatory attempt to counterbalance a tonic state of anhedonia, despite negative consequences [21]. This hypothesis has also been proposed for other typologies of addiction [22, 23].

The relationship between gambling disorder and obsessive-compulsive disorder has been studied from different perspectives. Most of the researches are related to the phenomenological aspects of these two disorders [24–26]. Some studies have concurrently compared PG and OCD from the personality perspective, showing similarities in personality dimensions and pointing out that PG and OCD patients share similar profiles [27]. Blaszczynski, in 1999 [28], evaluated the presence of obsessions and compulsions in subjects with PG using the Padua Inventory and highlighted specific results in obsessiveness in pathological gamblers when compared to control subjects. On the other side, the study of Won Kim and Grant [29] did not confirm the results, and other studies have reported that PG shares more similarities with SUDs than OCD [15, 30–32]. Further researches have also evidenced the presence of other dimensions, such as novelty seeking and self-transcendence [33, 34]. Moreover, a review of 2008, in an attempt to integrate the knowledge in the field of pathological gambling, proposed a new theoretical model of three specific subtypes of PG, which may be useful to find more appropriate treatments for the different subtypes. One of the three is the obsessive-compulsive subtype, distinct from the addictive subtype and the impulsive subtype. The authors have highlighted that the OC subtype includes around 20–25% of players, mostly women, who develop gambling behaviours in response to negative emotional states, suggesting that this type may respond better to treatment with antidepressants, SSRIs, and SNRIs, associated with psychotherapy [35]. A more recent study, conducted on an Italian sample, assessed the prevalence of the different subtypes among players, showing a good presence, but not predominant, of the OC subtype in the subject population; researchers have also considered a possible combination between the different subtypes, suggesting the utility of different treatments for each of them [36]. Differentiating into subtypes represents probably a correct way to assess substance dependences, as previously described in other studies [34, 37, 38].

The aims of our study were (1) to assess the prevalence of gambling behaviour in an Italian sample, considering the influence of sociodemographic variables, and (2) to assess the correlations between gambling behaviour and obsessive-compulsive features.

## 2. Methods and Procedure

### 2.1. Subjects

300 Italian adults were evaluated for gambling and obsessive-compulsive behaviours. The evaluation was carried out in Italy, more specifically in small towns in Lazio, Campania, and Sicily, mainly in coffee and tobacco shops where slot machines are located and there are state lottery offices. Participants were informed about the aims of the study and their participation was free. The questionnaires were anonymous and self-administered.

The study protocol complied fully with the guidelines of the Ethics Committee of the Cassino University of South Lazio and was approved by the Institutional Review Boards in accordance with local requirements. It was conducted in accordance with the Good Clinical Practice guidelines and the Declaration of Helsinki (1964) and subsequent revisions. After receiving information about the study, all the subjects provided written informed consent.

### 2.2. Assessment

Pathological gambling was evaluated with the* South Oaks Gambling Screen* (SOGS). Sociodemographic variables and obsessive-compulsive features were examined and evaluated with parts of the Cognitive Behavioural Assessment 2.0 (CBA 2.0).

The used scales used are summarised below.

The* South Oaks Gambling Screen* (SOGS) is a 20-item questionnaire based on DMS-III diagnostic criteria for pathological gambling. This is a self-administered questionnaire and appears to be a valid and reliable evaluation instrument for the rapid screening of pathological gambling [39]. The Cognitive Behavioural Assessment 2.0 (CBA 2.0) battery consists of ten schedules, each comprising items that explore a specific aspect of the subject, such as personal history, anxiety, some dimensions of personality, stress, fears, depression, and obsessions and compulsions [40].

The specific scales of* CBA 2.0* used in our study were schedule 1 and schedule 9.* Schedule 1* is a 25-item schedule and collects personal data. It is a sort of autobiographical folder that assesses the personal history of the subject. Moreover, schedule 1 investigates scholastic and academic history and current conditions of cohabitation [40].* Schedule 9* (MOCQ-R) is a reduced form of* Maudsley Obsessional-Compulsive Questionnaire* (MOCQ) [41]. MOCQ-R consists of 21 dichotomous items, split into three subscales, each comprising 7 items investigating the three main specifications of the obsessive-compulsive disorder: checking (behaviours related to repeated and unnecessary checking), cleaning (worries about hygiene, cleanliness, and unlikely contamination), and doubting-ruminating (recurrent doubts and intrusive thoughts) [40, 42, 43].

## 3. Data Analysis

The results were analysed using parametric and nonparametric statistics tests, with the SPSS program. When data were presented as frequencies of categories, we used the chi-square test to determine the significance of differences between two independent groups. When the studied variable had a normal distribution in the population from which the sample was extracted, we used Student's *t*-test to compare two independent means. When the studied continuous variable did not have a normal distribution, nonparametric tests were used. Baseline demographic and clinical features were compared across the sample using chi-square test for categorical variables and Kruskal Wallis test for continuous variables. Significance level was set at *P* < 0.05. Pearson's correlation coefficient was used to verify the correlations between SOGS and MOCQ-R. Primary outcome measures were obsessive-compulsive behaviours, for example, ritual of control (checking), rituals related to order and symmetry (ordering), ritual of washing and decontamination procedures (cleaning), and obsessive thoughts (obsessing). Secondary outcomes measures were aimed at differentiating between pathological and social gamblers, also identifying a range of problematic threshold with respect to gambling. In order to examine the structure of the SOGS and MOCQ-R, we used the principal components analysis (PCA) of the correlation matrix of the questions that compose the scales. In order to identify which questions were more represented by which component, we used as a criterion a factor loading greater than 0.4. For the inclusion of a question in the model to be submitted to the principal components analysis we used as a criterion the coefficient of determination (*R* ^²^) greater than 0.15.

## 4. Results

The sample consisted of 300 subjects aged between 18 and 65 years old (mean age: 36; SE: 0.76; SD: 13.18). Males were 174 (58%) and females 126 (42%). The choice of subjects was randomized. We firstly tested the reliability of the* South Oaks Gambling Screen* instrument. We obtained a high alpha of Cronbach (0.89). According to the scoring of the scale, respondents who obtain a score ranging from 0 to 2 are considered subjects with a good control of the gaming situation; respondents who obtain scores of 3 and 4 are classified as gamblers who are on a critical threshold; and finally those who score 5 or more are classified as pathological gamblers. Among these, scores higher than 5 indicate a serious problem with gambling. As a result of this classification and in relation to the scores obtained, the participants were divided into four score groups (1 = good control; 2 = critical threshold; 3 = problems with gambling; 4 = serious problems) and were considered under three main age groups with an interval of fifteen years each (18–33; 34–49; 50–65). As reported in Table 1, in the distribution of the total sample, 17.3% lied in the area characterized by a pathological relationship with gambling. Considering also those in the range of critical threshold with respect to gambling, we could identify a very large group of subjects (25.6%). Analysing the gender distribution in the groups with more severe problems, we recorded a significantly higher prevalence of men. The most involved age group in pathological levels of gambling was the first (18–33 years old), while the threshold cases belonged predominantly to the second age group (39–49 years old). The trends were the same for both males and females, although with a different proportion of subjects. The chi-square test allowed us to record significant associations of SOGS scores with the variables gender, age, marital status and education, with *P* < 0.05. We recorded significant differences in subjects belonging to the group of threshold issue with respect to gambling. It was preferably composed of single males, aged between 18 and 34, with high education.While in the group with serious problem on gambling significant difference was in the presence of single males, aged between 34–49. We then proceeded to analyse the scoring of the second instrument (*MOCQ-R*), after verifying the reliability of the three subscales, where the alphas obtained were, respectively, 0.74 (checking subscale), 0.53 (cleaning subscale), and 0.71 (doubt/rumination subscale).  Table 2 reports the distribution of cases in which manifestations indicated a clinically significant presence of obsessions and/or compulsions (score > cut-off percentiles, that is, 89, 89, and 94 for the three subscales and 94 for total score) and also the distribution of cases in which behaviours and concerns indicated a borderline psychological condition with pathological aspects (scores between seventy-fifth percentile and cut-off percentile). In the first age range (18–33), we observed obsessive-compulsive behaviours corresponding to values that exceed the cut-off score only among the women group (4 cases, *N* = 70), and they were specifically related to the areas of cleaning and doubt/rumination. Differently, taking into account the lower 75th percentile of the cut-off, we found 20 cases among men (*N* = 85) and 15 among women. In the men group, the top issues we identified were in the spheres of control and doubt/rumination, whereas for women it was the sphere of cleaning to be mainly involved. Switching to the second age group (34–49), we could note how situation was different: 8 cases among men, four of which in the sphere of cleaning, while no cases were reported for women. Considering also the 75th percentile of scores, male cases rose to 26, with a marked prevalence of issues related to control and doubt/rumination. In the third age group (44–65) the situation rebalanced: no cases over the cut-off for both genders; focusing on the 75th percentile, there was evidence of 4 cases for both males and females, and for the first time there were issues in the areas of checking and control for the female group. The total scores of MOCQ Index in the first group highlighted a transition among the cases of males belonging to the pathological area to an extensive portion in which obsessive and compulsive behaviours become issues of prevailing interest for the person; in men this fluctuated from 15.29% to 23.52%, while in women the variation ranged from 2.8% to 15.29%. In the second age group there were no notable changes through the scoring of total MOPQ Index, with 15% of cases with a marked tendency to obsessive and compulsive behaviours and compulsive thinking among men. In the third age group there were no significant changes as well; considering the 75th percentile there were no clinical cases and, respectively, 13.88% (men) and 20.68% (women) with a marked tendency to obsessive and compulsive behaviours and compulsive thinking.

Finally, we analysed the correlations between SOGS and MOPQ in order to further check the links of association between obsessive and compulsive behaviours in the management of dysfunctional situations with practices of gambling. As the calculation of scores for MOCQ is provided separately by age and sex, we verified separate correlations. The results reported in Table 3 indicate that, for the first age group (18–33), there was a slight negative correlation among males between the SOGS scores and scale of control (−0.21 significant at *P* < 0.05, two-tailed); for females there was an even slighter negative correlation with the scale of cleaning (−0.25) and the one of control (−0.25). Between the subscales of MOCQ, strong correlations were instead found, with a *P* < 0.01, and precisely between control and doubt *r* = .72 for males, *r* = .74 for females. among females it also emerged a correlation of .74 between cleaning and doubt, and *r* = 1.000 between cleaning and control. For the second age group (34–49) we found higher correlations between scores of SOGS and MOCQ. In females *r* = −.38 between cleaning and SOGS, while for males *r* = −.28 between control and SOGS, and *r* = −.30 for SOGS and Total MOCQ. Associations between subscales of the latter instrument were even higher. Also, *R* = .68, *P* < 0.05, between doubt and control for males; .56 and .84 were the associations between total MOCQ and cleaning and doubt, respectively. For females, control correlated with cleaning (*r* = .68, *P* < 0.05), and even highly with doubt (*r* = .77). A high correlation of 0.95 was reported also with total MOCQ. Switching to the third group (50–65) we found no correlations between SOGS and MOPQ. In this age range the excellent binding for women of the subscale of doubt with those of control (*r* = .75) and cleaning (*r* = .68) was confirmed; very high correlation was also with MOCQ total (*r* = .92). For men, the values tended to be lower between control and cleaning (*r* = .42) and with the doubt/rumination (*r* = .67), while we could still register robust associations with total MOCQ (*r* = .88, *r* = .89).

The chi-square test allowed us to record significant associations of MOCQ subscales with the variables of marital status and eventual cohabitation. In the first case the association was significant for all subscales of the MOCQ, with *P* < 0.05 for both women and men, greater for the first age group compared to the second and third; no significance was registered in regard to the variable of cohabitation/living alone.

## 5. Discussion

The study has certainly made it possible to identify within the sample a significant number of subjects who were excluded from the count of those with a disorder of the obsessive-compulsive spectrum by their behavioural characteristics of thought and actions. Correlation results between the means made us further reflect on the perspective from which obsessive-compulsive disorders and gambling are to be approached. The debate in the literature is still quite open and ranges from the model of gambling as a pathological behaviour (where obsessive-compulsive components play a core function) to the model of behavioural addictions. The controversy stems from the fact that often, from empirical evidence, both of the features that certainly belong to the spectrum of obsessive-compulsive disorder coexist in gamblers, as well as elements which may instead allow different theories. In our study, on one hand we discovered with certainty profiles of obsessive-compulsive disorder, but the association of correlation between the severity of the problem and the amplitude and frequency of obsessive-compulsive behaviours was negative. This suggests that, as the problem worsens, the gaming activity decreases the tendency to continuous checking, the emergence of doubts and the intrusive thoughts, or the compulsive rituals of cleaning. This tendency appears to be more pronounced in the second group (age 34–49), which is the one whose subjects showed the most serious problem with gambling. The explanation in this case may suggest on one hand that, in gambling approach, even in an elective manner, subjects with obsessive-compulsive inclinations immersed in the frantic repetition of sessions may play a privileged channel of repeated reassurance about their ability to control, in this case with a practice composed of short rhythmic sessions, and providing continuous feedback, repetition, and new refocusing to the subject that basically exorcises the fear of disintegration. On the other hand, the frenetic attempt to keep everything together and continuously allocated may fail after a certain extent. The obsessive-compulsive compensation gives way to the emergence of a genuine self-regulatory dysfunction, when neither the final obsessive control nor the intrusive thoughts nor the rituals can protect the subject.

The relationship established between the gambler and the gaming machines is certainly very close to a substance dependency. In this perspective, the discovery of a negative correlation with the spheres of control and cleaning makes sense, as the subject undergoes a real weakening of executive abilities and self-determination. This interpretation does not intend to exclude obsessive and dependent behaviours; it rather tries to catch a glimpse of the condition for the transition from one form to the other.

Data show actual critical issues related to gambling in the Italian context, especially in the provincial areas. In the 18- to 33-year-old group, besides the presence of clinically relevant situations related to obsessive compulsive-components, we noted a concentration and a widespread attitude towards these behaviours, although not yet patently pathological. Increasing behavioural problems linked to the need for resources, the growing debt load, the decreased attention while working and/or studying, the family issues, and the difficulties in the management of personal contacts appear to be increasingly related to the context of the game and an obsessive-compulsive disorder. The core question is a better understanding of the factors that contribute to maintaining the active participation of the gambler and his/her pervasive practice. Aim of this study was also to monitor the fluctuations in the emergence of critical cases according to different age distribution. As we can see from our results, the diffusion of problematic gambling among male population increases with age, for both serious and mild symptoms. Another aspect that deserves attention is the evaluation and mobilization of protection factors for the subject with gambling problems. The family still appears to be the main institution capable of providing not only emotional containment but also support to the increasing economic issues that recursively involve the gambler, who is no longer able to maintain the efficiency of the his/her self-regulatory resources. From this perspective, it becomes very important to have reliable tools for the detection and early evaluation of cases.

## 6. Conclusions

The results showed limited correlations between the instruments we used for assessment, but they have individually shown a good discriminative capacity and reliability in all the measurements. Through this study, it was possible to verify the significance and the pertinence of a combined use of two instruments for the measurement of the perception of gambling problems by patrons of lounges and bars equipped with slot machines, aiming at the simultaneous evaluation of the obsessive-compulsive components. In Italy, where the phenomenon has exponentially increased, partly because of strong interests (more or less legitimate) related to the field, it will be necessary to consistently keep monitored the fast evolving situation. Parallel to an accurate assessment, we must identify appropriate methods of intervention specific to the nature of involved individuals and their contexts, so that we can obtain incisive, and not just momentary, results. Another issue is the development of treatment options, also in light of the findings from the study that we conducted; it would be more appropriate to develop models that are able to balance both the pathophysiological aspect and the cognitive behaviour, in order to intervene in what appears to emerge as a nonhomogeneous syndrome.

According to our study results, the sample of gamblers we analysed does not belong to the sphere of obsessive-compulsive disorders, as previously described in clinical [44, 45] and neurobiological studies, showing differences between corticostriatal neural projections involved in OCD and gambling behaviours [46, 47]. Compulsive behaviours observed in PG are more similar to compulsivity observed in SUD, compared to typical compulsivity shown by OCD patients. This is in line with the approach recently proposed by DSM-5; however, further studies are needed in order to clarify the heterogeneous concept of compulsiveness [45].

In addition, the emergence of negative correlations between the tools leads to the hypothesis of an aggravation of gambling behaviours as a further form of deconstruction that predisposes a relationship of no control not oriented by a compulsive disorder, but by a more extensive self-regulatory dysfunction, which affects the subject's ability to evade the new structuring of a dependency relationship.

In conclusion, our findings confirm the validity of the model proposed by DSM-5 for the classification of PG and suggest the importance of investing in treatments similar to those used for substance use disorders, rather than for obsessive-compulsive disorders [48–50].

## Figures and Tables

**Table 1:** Scores of SOGS with reference to age.

				Class of age			
				18–33	34–49	50–65	Total
Males	Score ranges	1.00	Count	54	29	28	111
			% within score range	48.6%	26.1%	25.2%	100.0%
			% within class of age	63.5%	54.7%	77.8%	63.8%
			% within total	31.0%	16.7%	16.1%	63.8%
		2.00	Count	8	10	2	20
			% within score range	40.0%	50.0%	10.0%	100.00%
			% within class of age	9.4%	18.9%	5.6%	11.5%
			% within total	4.6%	5.7%	1.1%	11.5%
		3.00	Count	16	12	6	34
			% within score range	47.1%	35.3%	17.6%	100.00%
			% within class of age	18.8%	22.6%	16.7%	19.5%
			% within total	9.2%	6.9%	3.4%	19.5%
		4.00	Count	7	2	0	9
			% within score range	77.8%	22.2%	0.0%	100.00%
			% within class of age	8.2%	3.8%	0.0%	5.2%
			% within total	4.0%	1.1%	0.0%	5.2%
			**Total count **	**85**	**53**	**36**	**174**
			**% within total age**	**48.9%**	**30.5%**	**20.7%**	**100.00%**

Females	Score ranges	1.00	Count	62	23	27	112
			% within score range	55.4%	20.5%	24.1%	100.00%
			% within class of age	88.6%	85.2%	93.1%	88.9%
			% within total	49.2%	18.3%	21.4%	88.9%
		2.00	Count	3	2	0	5
			% within score range	60.0%	40.0%	0.0%	100.0%
			% within class of age	4.3%	7.4%	0.0%	4.0%
			% within total	2.4%	1.6%	0.0%	4.0%
		3.00	Count	4	0	2	3
			% within score range	66.7%	0.0%	33.3%	100.0%
			% within class of age	5.7%	0.0%	6.9%	4.8%
			% within total	3.2%	0.0%	1.6%	4.8%
		4.00	Count	1	2	0	3
			% within score range	33.3%	66.7%	0.0%	100.0%
			% within class of age	1.4%	7.4%	0.0%	2.4%
			% within total	0.8%	1.6%	0.0%	2.4%
			**Total count**	**70**	**27**	**29**	**126**
			**% within total age**	**55.6%**	**21.4%**	**23.0%**	**100.00%**

Total	Score ranges	1.00	Count	116	52	55	223
			% within score range	52.0%	23.3%	24.7%	100.0%
			% within class of age	74.8%	65.0%	84.6%	74.3%
			% within total	38.7%	17.3%	18.3%	74.3%
		2.00	Count	11	12	8	25
			% within score range	44.0%	48.0%	8.0%	100.0%
			% within class of age	7.1%	15.0%	12.3%	8.3%
			% within total	3.7%	4.0%	2.7%	8.3%
		3.00	Count	20	12	8	40
			% within score range	50.0%	30.0%	20.0%	100.0%
			% within class of age	12.9%	15.0%	12.3%	13.3%
			% within total	6.7%	4.0%	2.7%	13.3%
		4.00	Count	8	4	0	12
			% within score range	66.7%	33.3%	0.0%	100.0%
			% within class of age	5.2%	5.0%	0.0%	4.0%
			% within total	2.7%	1.3%	0.0%	4.0%
			**Total count**	**155**	**80**	**65**	**300**
			**% within total age**	**51.7%**	**26.7%**	**21.7%**	**100.0%**

Score ranges: 1 = good control (scores from 0 to 2); 2 = critical threshold (scores from 3 to 4); 3 = problem with gambling (scores from 5 to six); 4 = serious problem (scores higher than six). Count: number of subjects within the range.

**Table 2:** Scoring of MOCQ-R.

Group of age	*N*.	Sex	MOCQ-R1-R2-R3	Subjects with values > cut-off	Subjects with values >75° percentile
18–33	85	Males	R1 control	0	9
			R2 cleaning	0	3
			R3 doubt/rumination	0	8
			Total Index MOCQ	13	20
			% total index	*15.29% *	*23.52% *
	70	Females	R1 control	0	2
			R2 cleaning	2	10
			R3 doubt/rumination	2	3
			Total Index MOCQ	2	13
			% total index	*2.8% *	*15.29% *

34–49	53	Males	R1 control	2	10
			R2 cleaning	4	4
			R3 doubt/rumination	2	12
			Total Index MOCQ	0	8
			% total index	*0.0% *	*15% *
	27	Females	R1 control	0	0
			R2 cleaning	0	0
			R3 doubt/rumination	0	3
			Total Index MOCQ	0	3
			% total index	*0.0% *	*5.66% *

50–65	36	Males	R1 control	0	2
			R2 cleaning	0	0
			R3 doubt/rumination	0	2
			Total Index MOCQ	0	5
			% total index	*0.0% *	*13.88% *
	29	Females	R1 control	0	4
			R2 cleaning	0	0
			R3 doubt/rumination	0	0
			Total Index MOCQ	0	6
			% total index	*0.0% *	*20.68% *

MOCQ-R investigates obsessive-compulsive behaviors and problems, not symptoms or personality traits, and the total score estimates the extent and severity of these problems. MOCQ-R1 indicates specific behaviors and concerns related to repeated and unnecessary controls; MOCQ-R2 indicates problems related to hygiene and cleanliness, as well as concerns related to unlikely infection and contamination; MOCQ-R3 indicates recurrent and intrusive thoughts and unpleasant and persistent doubts; Total Index MOCQ: indication of the presence of obsessive-compulsive disorders.

**Table 3:** Correlations between SOGS and MOCQ-R.

			Scores of SOGS	Control MOCQ-R1	Cleaning MOCQ-R2	Doubt MOCQ-R3
Control males	Group of age 18–33	Pearson correlation Sig. (2-tailed) *N*	**−.219** ∗ .044 85			**.725** ∗∗ .000 85
Control females		Pearson correlation Sig. (2-tailed) *N*	**−.250** ∗ .037 70		**1.000** ∗∗ .000 70	
Cleaning males		Pearson correlation Sig. (2-tailed) *N*				
Cleaning females		Pearson correlation Sig. (2-tailed) *N*	**−.250** ∗ .037 70	**1.000** ∗∗ .000 70		
Doubt males		Pearson correlation Sig. (2-tailed) *N*		**.725** ∗∗ .000 85		
Doubt females		Pearson correlation Sig. (2-tailed) *N*		**.741** ∗∗ .000 70	**.741** ∗∗ .000 70	
Total MOCQ males		Pearson correlation Sig. (2-tailed) *N*		**.846** ∗∗ .000 85	**.463** ∗∗ .000 85	**.850** ∗∗ .000 85
Total MOCQ females		Pearson correlation Sig. (2-tailed) *N*		**.904** ∗ .044 70	**.904** ∗ .044 70	**.888** ∗∗ .000 70

Control males	Group of age 34–49	Pearson correlation Sig. (2-tailed) *N*	**−.286** ∗ .038 53			**.682** ∗∗ .000 53
Control females		Pearson correlation Sig. (2-tailed) *N*			**.680** ∗∗ .000 27	
Cleaning males		Pearson correlation Sig. (2-tailed) *N*				
Cleaning females		Pearson correlation Sig. (2-tailed) *N*	**−.389** ∗ .045 27	**.680** ∗∗ .000 27		
Doubt males		Pearson correlation Sig. (2-tailed) *N*		**.682** ∗∗ .000 53		
Doubt females		Pearson correlation Sig. (2-tailed) *N*		**.779** ∗∗ .000 27		
Total MOCQ males		Pearson correlation Sig. (2-tailed) *N*	**−.302** ∗ .028 53	**.880** ∗∗ .000 53	**.567** ∗∗ .000 53	**.841** ∗∗ .000 53
Total MOCQ females		Pearson correlation Sig. (2-tailed) *N*		**.955** ∗∗ .000 27	**.779** ∗∗ .000 27	**.842** ∗∗ .000 27

Control males 50–65	Group of age 50–65	Pearson correlation Sig. (2-tailed) *N*			**.429** ∗∗ .009 36	**.676** ∗∗ .000 35
Control females		Pearson correlation Sig. (2-tailed) *N*			**.650** ∗∗ .000 29	
Cleaning males		Pearson correlation Sig. (2-tailed) *N*		**.429** ∗∗ .009 36		**.449** ∗∗ .007 35
Cleaning females		Pearson correlation Sig. (2-tailed) *N*		**.650** ∗∗ .000 29		
Doubt males		Pearson correlation Sig. (2-tailed) *N*		**.676** ∗∗ .000 35	**.449** ∗∗ .007 35	
Doubt females		Pearson correlation Sig. (2-tailed) *N*		**.751** ∗∗ .000 29	**.683** ∗∗ .000 29	
Total MOCQ males		Pearson correlation Sig. (2-tailed) *N*		**.883** ∗∗ .000 35	**.666** ∗∗ .000 35	**.898** ∗∗ .000 35
Total MOCQ females		Pearson correlation Sig. (2-tailed) *N*		**.925** ∗∗ .000 29	**.828** ∗∗ .000 29	**.914** ∗∗ .000 29

*Correlation is significant at the 0.05 level (tailed).

**Correlation is significant at the 0.01 level (2-tailed).
